# *Astragali Radix* Contributes to the Inhibition of Liver Fibrosis via High-Mobility Group Box 1-Mediated Inflammatory Signaling Pathway

**DOI:** 10.1155/2021/5574010

**Published:** 2021-03-13

**Authors:** Jianxia Wen, Dan Wang, Jian Wang, Ruilin Wang, Shizhang Wei, Yanling Zhao

**Affiliations:** ^1^Department of Pharmacy, The Fifth Medical Center of Chinese PLA General Hospital, Beijing 100039, China; ^2^College of Pharmacy, Chengdu University of Traditional Chinese Medicine, Chengdu 611137, China; ^3^Chongqing Academy of Chinese Materia Medica, Chongqing 400065, China; ^4^Clinical Laboratory Medicine Center, The Fifth Medical Center of Chinese PLA General Hospital, Beijing 100039, China

## Abstract

*Astragali Radix* (AR), the dried root of *Astragali Radix membranaceus* (Fisch.) Bge. or *Astragali Radix membranaceus* (Fisch.) Bge. var. mongholicus (Bge) Hsiao, is a commonly used traditional Chinese medicine for the treatment of liver diseases. This study aimed to comprehensively evaluate the pharmacological action and explore the potential mechanism of AR on liver fibrosis. Rats were administered with carbon tetrachloride for eight weeks, followed by oral treatment with AR for six weeks. The efficacy was confirmed by measuring liver function and liver fibrosis levels. The underlying mechanisms were explored by detecting the expression of related proteins. AR significantly decreased the serum levels of alanine aminotransferase (ALT), aspartate aminotransferase (AST), collagen IV (COL-IV), hyaluronic acid (HA), laminin (LN), and precollagen type III (PCIII). In addition, AR inhibited the deposition of collagen and the activation of hepatic stellate cells. Those data strongly demonstrated that AR alleviated liver fibrosis by CCl_4_. In order to illustrate the potential inflammatory, the mRNA levels of IL-6, TNF-*α*, and IL-1*β* were detected. Subsequently, immunohistochemistry analysis was performed to further verify the expression of type I collagen. Finally, the expression of key proteins in the inflammatory signaling pathway was detected. AR significantly reduced the expression of high-mobility group box 1 (HMGB1), TLR4, Myd88, RAGE, and NF-*κ* B p65 genes and proteins. In addition, western blotting showed AR decreased the protein expression of RAGE, p-MEK1/2, p-ERK1/2, and p-c-Jun. Taken together, our data reveal that AR significantly inhibits liver fibrosis by intervening in the HMGB1-mediated inflammatory signaling pathway and secretion signaling pathway. This study will provide valuable references for the in-depth research and development of *Astragali Radix* against liver fibrosis.

## 1. Introduction

Liver fibrosis is a complex pathological process and an intermediate link between various liver diseases including cirrhosis and liver cancer [1, 2]. And prevention of malignant liver diseases by inhibiting the occurrence and development of liver fibrosis has attracted worldwide attention [3]. Numerous studies have revealed the pathogenesis of liver fibrosis, such as inflammatory response, activation of hepatic stellate cells, and excessive deposition of extracellular matrix, but it has not yet been transformed into an effective and powerful treatment [4, 5]. There is still a lack of safe and effective means of treating liver fibrosis [6, 7].

It is necessary to develop new drugs for treating liver fibrosis [7]. Traditional Chinese medicine has potential advantages in the treatment of chronic diseases, so we tried to find effective drugs for the treatment of liver fibrosis. *Astragali Radix* (AR), the dried root of *Astragali Radix membranaceus* (Fisch.) Bge. or *Astragali Radix membranaceus* (Fisch.) Bge. var. mongholicus (Bge) Hsiao, is a commonly used traditional Chinese medicine for the treatment of liver diseases [8]. And it has been proved that AR possesses hepatoprotective and anti-inflammatory properties and has been shown to exhibit immunomodulating and antioxidant activity, among others [9]. In traditional Chinese formula, AR is often used for the treatment of liver fibrosis [10]. Although the protective effects of AR on liver injury and related mechanisms have been reported [9, 11], the studies on the effect and mechanism of AR on liver fibrosis are still lacking. More importantly, toxicological study has confirmed the high safety of AR. No significant toxicity or side effects were observed when the dose administered to rats was set to 39.9 g/kg [12].

Experimental and clinical studies have found that the high-mobility group box-1 (HMGB1), recently discovered damage-associated molecular patterns (DAMPs), is substantially enriched in patients with hepatic fibrosis [13, 14]. HMGB1 is a late inflammatory mediator that plays an important role in the pathogenesis of many chronic inflammations, which is the main cause of liver fibrosis [15]. There is evidence that HMGB1 can directly stimulate hepatic stellate cells (HSCs) activation to accelerate the development of liver fibrosis [16]. More importantly, toll-like receptor 4 (TLR4) and the receptor of advanced glycation end products (RAGE) are the main receptors of HMGB1 [15]. It is reported that the binding of extracellular HMGB1 to TLR4 could activate NF-*κ*B signaling to induce inflammation and aggravate liver fibrosis. Studies have shown that extracellular HMGB1 binding to RAGE could phosphorylate c-Jun to induce a significant increase in type I collagen secretion [16, 17], which directly accelerates the development of liver fibrosis (c-Jun is a component of the heterodimeric AP1 and is a potent transactivating factor for the *collagen1a1* and *collagen1a2* genes [17, 18]).

Based on previous studies, this study aims to provide answers to the following questions: Does AR have therapeutic effect on carbon tetrachloride- (CCl_4_-) induced liver fibrosis in rats? What is the underlying mechanism? Is the mechanism related to downregulation of HMGB1?

## 2. Materials and Methods

### 2.1. Reagents

In this study, colchicine was purchased from Xishuangbanna Banna Pharmaceutical Co. Ltd. (Yunnan, China). CCl_4_ was supplied by Tianjin Guangfu Chemical Research Institute (Tianjin, China). Alanine amino transferase (ALT, Cat. No.: 20180627), aspartate amino transferase (AST, Cat. No.: 20180607), collagen IV (COL-IV, Cat. No.: 201809), hyaluronic acid (HA, Cat. No.: 201808), laminin (LN, Cat. No.: 201809), and precollagen type III (PC III, Cat. No.: 201809) detection kits were provided by Nanjing Jiancheng Bioengineering Institute (Nanjing, China). The antibodies of phospho-c-Jun, phospho-MEK1/2, HMGB1, *α*-SMA, phospho-ERK1/2, and ERK1/2 were bought from Cell Signaling Technology (United States). The antibodies of MEK1/2 and COL-I were purchased from Abcam (United States) and the antibodies of RAGE and GAPDH were bought from ABclonal (China).

### 2.2. Plant Material

AR (the dried root of *AR membranaceus* (Fisch.) Bge.) was supplied by Beijing Lvye Pharmaceutical Co. Ltd. (Beijing, China). Ten times of water was used for the first extraction of AR for 2 h. Then, eight times of water was used to extract for 1.5 hours. The extract of *Astragalus membranaceus* was dried into powder by freeze vacuum drying oven. The final yield was about 40.08%. The Q-TOF LC/MS analysis showed that the main components of AR extract included 3,4-dihydroxybenzyl aldehyde (6.17%), calycosin-7-glucoside (0.69%), armillaripin (10.31%), rhodomollein III (7.05%), phthalic anhydride (1.76%), clavatine (6.46%), pterosin B (7.46%), D-cathinone (2.65%), formononetin (15.4%), Andrographolide (2.57%), and anemarrhenasaponin-I (4.55%) [19].

### 2.3. Animals and Administration

A total of 36 male Sprague Dawley rats weighting 180–200 g were obtained from the China Food and Drug Certification Research Institute (Permission No. SCXK (Jing)-2014-0013). Those rats were housed in the Central Animal Laboratory of the Fifth Medical Center of Chinese PLA General Hospital. The animals were maintained under controlled conditions of temperature 25°C ± 2°C, humidity 55% ± 5%, and with light and dark cycles.

All the animals were randomly divided into six groups with six rats in each group. Olive oil diluted CCl_4_ in a ratio of 1 : 1. The model group, AR (2.7, 5.4, 10.8 g/kg/d) groups, and colchicine (as a positive control group) groups were given CCl_4_ diluent (2 mL/kg) twice a week for 8 weeks [19]. At the same time, the control group was intraperitoneally injected with the same dose of olive oil. Eight weeks later, the AR (2.7, 5.4, and 10.8 g/kg/d) groups and colchicine group (0.2 mg/kg/d) were given the corresponding concentration of drugs [20]. The control group and model group were given equal volume of distilled water. All rats were treated by intragastric administration once a day for 6 weeks. All rats were deprived of food for 24 h before the last treatment but allowed free access to water. The animals were sacrificed 2 h after the last treatment and their liver and serum were harvested for the further studies. The blood samples were centrifuged at 3500 rpm for 15 min. The liver tissue was divided into 3 pieces and placed in the sample bottle. Serum and liver tissue were stored at −80°C. All animal experiments were approved by the Ethical Committee of the Fifth Medical Center of Chinese PLA General Hospital in China.

### 2.4. Biochemical Assay

The serum levels of ALT, AST, COL-IV, HA, LN, and PC III were measured. In brief, according to the manufacturer's protocols, the samples, 2,4-dinitrophenylhydrazine solution, and sodium hydroxide solution were added in turn. The final color was read at 510 nm, and the serum ALT and AST levels were determined according to the OD value. Similarly, according to the manufacturer's instructions, add the reagents in the kit in turn, read the final color at 450 nm, and obtain the serum levels of COL-IV, HA, LN, and PC III according to the OD value.

### 2.5. Histological Examination

Liver tissue was fixed in 10% neutral buffered formalin for more than 48 h. All the livers were eluted with gradient ethanol solution and fixed and embedded in paraffin. Tissue sections about 4-5 *μ*m in thickness were cut and stained with hematoxylin eosin (H&E) and Masson. The pathological changes in the liver tissues were observed under a Nikon microscope (Nikon Instruments Inc., Japan).

### 2.6. Quantitative Reverse Transcription Polymerase Chain Reaction (RT-PCR) Analyses

Quantitative RT-PCR was used to detect the mRNA expression of IL-6, TNF-*α*, IL-1*β*, HMGB1, TLR4, Myd88, and RAGE in liver tissues. Total RNA was extracted from 90 mg frozen liver tissue by Trizol reagent (Life Technologies, CA, USA). The absorbance of the extracted mRNA was detected by spectrophotometer at 260 nm and 280 nm, respectively. The mRNA with purity (A260/A280) between 1.8 and 2.2 was selected as the qualified product for the follow-up study. RNA (2 *μ*g) was transcribed into cDNA by PrimerScript RT regent kit (Thermo Fisher Scientific, MA, USA). The cDNA was subsequently subjected to PCR amplification by ABI 7500 Real-Time PCR machine (Applied Biosystems Inc, Carlsbad, CA, USA). Data analysis was performed by the 2^−△△CT^ method. The primers are listed in Table 1.

### 2.7. Western Blotting

The mixture of ice-cold radio immunoprecipitation assay (RIPA) lysis buffer, phenylmethylsulfonyl fluoride (PMSF), and protein phosphatase inhibitor was used for protein extraction. Liver tissue (80 mg) was homogenized and lysed in the mixture, and then centrifuged at 12000 × g and 4°C for 10 min. The supernatant was collected and BCA Protein Assay kit (Beijing Solarbio Science & Technology Co., Ltd. Beijing, China) was used to detect the total protein concentration. All protein samples were levelled to the same concentration. Then, reducing Laemmli SDS sample buffer was added and mixed well, and boiled in boiling water for 5 minutes. The prepared samples were western blotted with 10% sodium dodecyl sulfate-polyacrylamide gel electrophoresis (SDS-PAGE) and the blot was transferred to the polyvinylidene fluoride (PVDF) membrane. The PVDF membrane was sealed in 5% skimmed milk at room temperature for 2 hours and then placed in a mixture of 5% skimmed milk, Tris-buffered saline (TBS), and 0.05% Tween 20 with the corresponding primary antibody, and incubated overnight at 4°C. Antibodies, including rabbit Anti-NF-*κ*B p65 (Cell Signaling Technology, dilution: 1 : 1000), RAGE (ABclonal technology, dilution: 1 : 1000), phospho-MEK1/2 (Cell Signaling Technology, dilution: 1 : 2000), MEK1/2 (Abcam, dilution: 1 : 10000), phospho-ERK1/2 (Cell Signaling Technology, dilution: 1 : 1000), ERK1/2 (Cell Signaling Technology, dilution: 1 : 10000), phospho-c-Jun (Abcam, dilution: 1 : 500), and GAPDH (ABclonal technology, dilution: 1 : 10000), were used at this step. GAPDH was used as control for total protein extract. After incubation with appropriate secondary antibody for 2 hours at room temperature, the membrane was washed 3 times in TBS with 0.05% Tween 20 for 5 minutes each. Finally, the chemiluminescence system was used to measure immunoreactive proteins. All the western blotting was repeated 3 times.

### 2.8. Immunohistochemistry Analysis

To evaluate the effects of AR on the expression of *α*-SMA, HMGB1, and collagen I in liver tissue of fibrosis rats, immunohistochemical analysis was performed as described previously [21]. Paraffin sections of liver tissues were put in xylene and gradient ethanol for deparaffinization and dehydration, and then the sections were put into citric acid antigen retrieval solution for antigen retrieval. The sections were treated with 3% hydrogen peroxide solution to block endogenous peroxidase. The sections were then blocked with 1% BSA for 30 minutes at room temperature. Subsequently, the sections were incubated with *α*-SMA (Cell Signaling Technology, dilution: 1 : 100), Rabbit Anti-HMGB1 (Cell Signaling Technology, dilution: 1 : 200), and collagen type I (Abcam, dilution: 1 : 200) in a PBS solution at 4°C overnight. After incubating the sections with appropriate secondary antibodies for 1 hour at room temperature, they were stained with DAB staining solution and hematoxylin, and finally the slides were dehydrated and mounted. Nikon microscope (Nikon Instruments Inc., Japan) and NIS-Elements (F 4.0 version, Japan) software were used to observe the sections. Finally, Image-Pro Plus (version 6.0, Media Cybemetics, INC., Rockville, MD, USA) software was used to calculate and analyze the average integrated optical density (IOD) of the positive area of the immunohistochemical reaction image.

### 2.9. Statistical Analysis

Results were expressed as mean ± standard deviation (SD). Data were evaluated by one-way ANOVA and Duncan's multirange test with the SPSS computer program (version 20.0, SPSS Inc., Chicago, IL, USA). *P* < 0.05 was considered statistically significant and *P* < 0.01 was considered highly significant.

## 3. Results

### 3.1. AR Has Therapeutic Effect on CCl_4_-Induced Liver Injury

**Figure 1:**
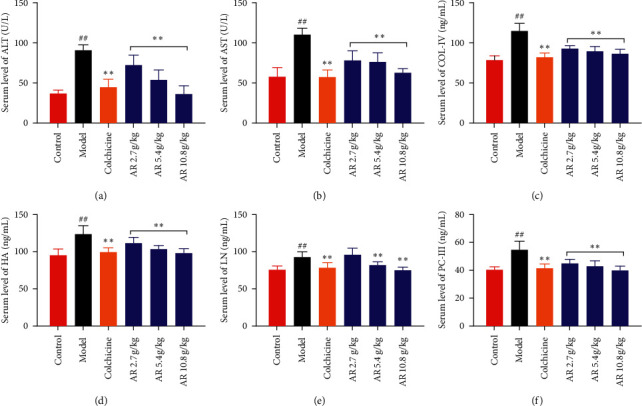
Effect of AR on CCl_4_-induced liver fibrosis in rats in different groups. (a) Serum level of ALT, (b) serum level of AST, (c) serum level of COL-IV, (d) serum level of HA, (e) serum level of LN, and (f) serum level of PC-III. Values are expressed as mean ± SD (*n* = 6). ^#^*P* < 0.05 and ^##^*P* < 0.01 when compared with the control group. ^*∗*^*P* < 0.05 and ^*∗∗*^*P* < 0.01 when compared with the model group.

As shown in Figures 1(a) and 1(b), the levels of ALT and AST in rats in the model group were significantly increased (*P* < 0.01) compared with those in the control group. And serum ALT and AST concentrations were significantly reduced after oral administration of AR 5.4 g/kg and 10.8 g/kg (*P* < 0.01). Subsequently, the serum levels of COL-IV, HA, LN, and PC III which could reflect the degree of hepatic fibrosis were examined [22]. As shown in Figures 1(c)–1(f), the serum levels of COL-IV, HA, LN, and PC III in the model group were significantly higher than those in the control group (*P* < 0.01). The inhibitory effect of AR on the levels of COL-IV, HA, LN, and PC III is dose-dependent. In particular, the serum levels of COL-IV, HA, LN, and PC III were significantly decreased in the 10.8 g/kg AR group (*P* < 0.01).

### 3.2. The Effect of AR on Liver Histopathological Changes and Liver Fibrosis

**Figure 2:**
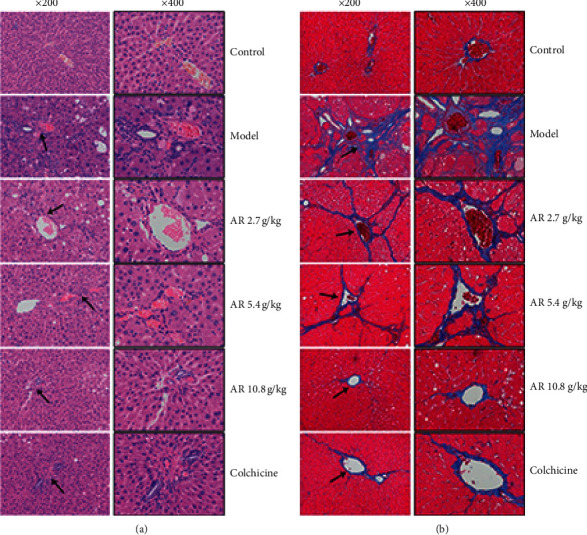
Histopathological observation of AR on improvement of liver fibrosis in rats. (a) Hematoxylin and eosin- (HE-) stained liver section in six groups (original magnification, ×200, ×400). (b) Histological examination of liver section with Masson stain (original magnification, ×200, ×400). Blue areas show collagen fibers and damaged liver tissue.

Then, hematoxylin-eosin (HE) staining and Masson staining were used to observe the pathological changes of liver tissue [21, 23]. The results of HE showed that the liver tissue structure of the control group was normal without morphological changes. The long-term CCl_4_-treated rats showed partial necrotic in their liver accompanied by severe steatosis and inflammatory cell infiltration around the central vein of hepatic lobules. In contrast, the degree of liver damage, steatosis, and inflammatory infiltration in the AR treatment groups were reduced to varied degrees in a dose-dependent manner (Figure 2(a)). In the liver section of Masson staining, the blue area represented the deposited collagen. The results show that a large amount of collagen was deposited around the hepatic sinus of the model group. AR can effectively reduce collagen deposition and improve liver fibrosis (Figure 2(b)). The above results confirmed that the CCl_4_-induced rat liver fibrosis model was successful, and AR could effectively ameliorate liver fibrosis in rats.

### 3.3. Inflammatory Response in Rats Induced by CCl_4_

**Figure 3:**
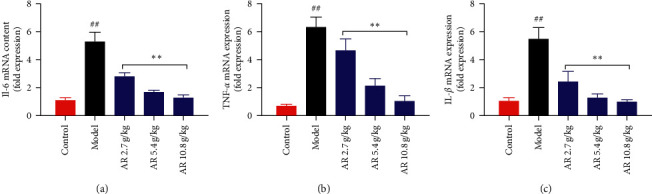
Effect of AR on inflammatory factors levels in liver fibrosis induced by CCl_4_. (a) IL-6 mRNA level. (b) TNF-*α* mRNA level. (c) IL-1*β* mRNA level. Values are expressed as mean ± SD (*n* = 6). ^#^*P* < 0.05 and ^##^*P* < 0.01 when compared with the control group. ^*∗*^*P* < 0.05 and ^*∗∗*^*P* < 0.01 when compared with the model group.

Clinical data suggest that inflammation is a key factor in the progression of liver fibrosis [24]. IL-6, as a classic proinflammatory cytokine biomarker, is used for clinical diagnosis of chronic liver fibrosis [25]. TNF-*α* can promote the survival of activated HSCs and promote the development of liver fibrosis [26]. It is reported that the serum IL-1*β* in patients with liver fibrosis caused by schistosomiasis is significantly increased [24]. Therefore, we detected the expression of IL-6, TNF-*α*, and IL-1*β* in rats by quantitative RT-PCR. The mRNA levels of IL-6, TNF-*α*, and IL-1*β* were increased remarkably in the CCl_4_-induced group compared with the control group (*P* < 0.01, Figure 3). After treatment with AR, the expression of related genes decreased in a dose-dependent manner (*P* < 0.05, *P* < 0.01). The results showed that AR had therapeutic effect on CCl_4_-induced liver inflammation in rats.

### 3.4. AR Downregulates CCl_4_-Induced Liver Inflammation

**Figure 4:**
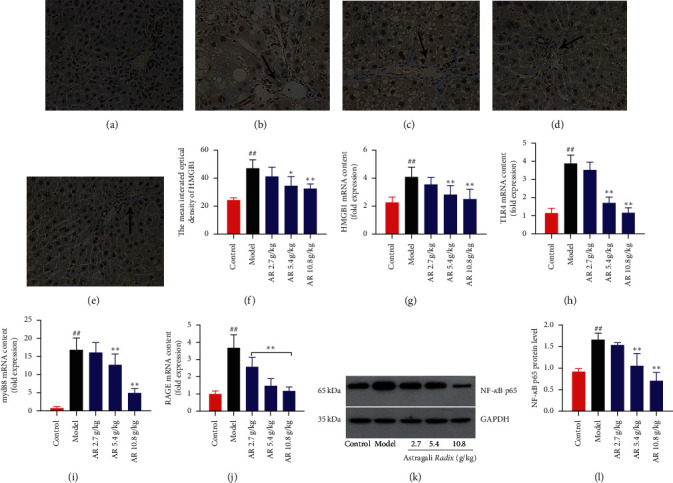
Effect of AR on HMGB1/TLR4/NF-*κ*B signaling pathway in liver fibrosis induced by CCl_4_. (a–e) Immunohistochemistry analysis for the effect of AR on HMGB1 localization (original magnification, ×400; *n* = 3; (a) control group; (b) model group; (c) 2.7 g/kg AR group; (d) 5.4 g/kg AR group; (e) 10.8 g/kg AR group). (f) The mean integrated optical density of HMGB1. (g) HMGB1 mRNA level. (h) TLR4 mRNA level. (i) Myd88 mRNA level. (j) RAGE mRNA level. (k) Western blot images of NF-*κ*B p65. (l) NF-*κ*B p65 protein level. Values are expressed as mean ± SD (*n* = 6). ^#^*P* < 0.05 and ^##^*P* < 0.01 when compared with the control group. ^*∗*^*P* < 0.05 and ^*∗∗*^*P* < 0.01 when compared with the model group.

Literature research found that HMGB1, a cytokine abundantly enriched in patients with liver fibrosis, mediated TLR4//NF-*κ*B signaling pathway is one of the important pathways for liver aseptic inflammation. Therefore, we tested the expression levels of HMGB1, TLR4, Myd88, and NF-*κ*B p65, which are the key targets of this signaling pathway in liver fibrosis rats. Quantitative RT-PCR and immunohistochemistry (IHC) methods were used to detect the expression and subcellular localization of HMGB1. As shown in Figures 4(a)–4(e), in the control group, HMGB1 was mainly located in the nucleus of hepatocytes and rarely in the cytoplasm or sinusoids. In the model group, the greater HMGB1 immunoreactivity was observed in the periportal and bridge fibrosis areas in the liver. In addition, computer-assisted semiquantitative analysis showed that AR treatment alleviated the expression of HMGB1 compared with the model group (Figure 4(f), *P* < 0.05, *P* < 0.01).

Furthermore, the mRNA expression of HMGB1 was also quantified by using the RT-PCR method. The results showed that high dose of AR could significantly reduce the mRNA expression of HMGB1, which is consistent with the result of the IHC method. Those results proved that AR could reduce the expression of HMGB1 (Figure 4(g)). Subsequently, quantitative RT-PCR was used to detect the TLR4, Myd88, and RAGE mRNA levels in the model group, which were significantly higher than those in the control group (Figures 4(h)–4(j), *P* < 0.01). After treatment with AR, the expression of related genes decreased in a dose-dependent manner (*P* < 0.05, *P* < 0.01). Western blotting was used to detect the expression level of NF-*κ*B p65 in the liver of rats with liver fibrosis. As shown in Figures 4(k)–4(l), the immunoblotting band of NF-*κ*B p65 in the model group was significantly thicker than that of the control group, and the statistical results showed significant differences (*P* < 0.01). After AR treatment, the expression of NF-*κ*B p65 decreased in a dose-dependent manner, especially in the 5.4 g/kg AR group and 10.8 g/kg AR group (*P* < 0.01). These results suggest that AR may improve liver inflammation by downregulating HMGB1/TLR4//NF-*κ*B signaling pathway.

### 3.5. AR Inhibits the Activation of HSCs

**Figure 5:**
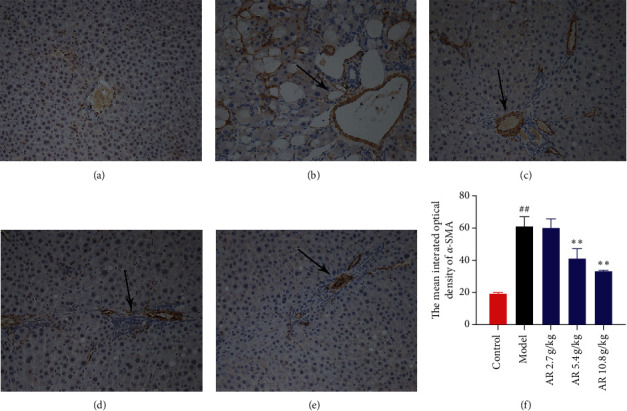
Immunohistochemistry analysis for the effect of AR on *α*-SMA localization (original magnification, ×200; *n* = 3; (a) control group; (b) model group; (c) 2.7 g/kg AR group; (d) 5.4 g/kg AR group; (e) 10.8 g/kg AR group). (f) Quantitative analysis of *α*-SMA localization by immunohistochemistry.

HSCs are the core cells of liver fibrosis. It is reported that HMGB1 can directly activate HSCs [16]. Therefore, we speculate that AR can play a protective role in liver fibrosis by reducing the activation of HSCs. *α*-smooth muscle actin (*α*-SMA) is a special marker of activated HSCs [27]. Here, the ratio of activated HSCs was measured by using IHC method through detecting *α*-SMA in the liver tissues (Figure 5). In the control group, only a small amount of *α*-SMA-positive cells was found in the portal area, which might be the hepatic arteries. More *α*-SMA-positive cells were observed in the CCl_4_-induced group compared with control group, and the *α*-SMA-positive cells were significantly reduced after AR treatment. By computer-assisted semiquantitative analysis, the mean integrated optical density (IOD) of the *α*-SMA-positive area of the AR treatment group was confirmed to be significantly reduced in a dose-dependent manner compared to model group (Figure 5, *P* < 0.01). Those results revealed that AR could reduce the activation of HSCs, a key cell of liver fibrosis.

### 3.6. AR Has an Effect on the Type 1 Collagen Secretion Signaling Pathway Mediated by HMGB1

**Figure 6:**
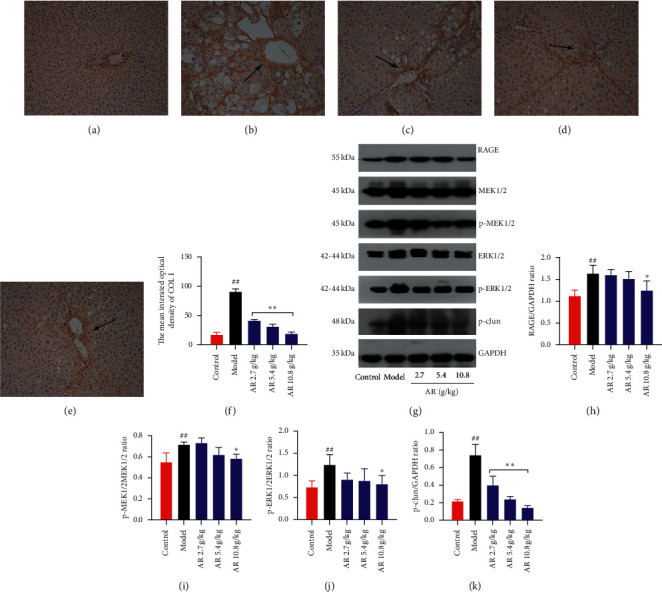
Effect of RA on type 1 collagen level and HMGB1/RAGE/c-Jun pathway in liver fibrosis induced by CCl_4_. (a–e) Immunohistochemistry analysis for the effect of AR on type 1 collagen expression (original magnification, ×200; *n* = 3; (a): control group; (b): model group; (c): 2.7 g/kg AR group; (d) 5.4 g/kg AR group; (e) 10.8 g/kg AR group.) (f) The mean integrated optical density of COL-I. (g) Western blot images of RAGE, MEK1/2, p-MEK1/2, ERK1/2, p-ERK1/2, and p-cJun. (h) RAGE protein level. (i) p-MEK1/2 protein level. (j) p-ERK1/2 protein level. (k) p-cJun protein level.

Next, IHC method was used to detect the expression of collagen type I. In Figures 6(a)–6(e), type I collagen deposition around the hepatic lobular portal vein in rats treated with CCl_4_ alone was significantly increased. After treatment with AR, the expression and deposition of type I collagen in the liver were significantly inhibited, especially in the group with the dose of 10.8 g/kg. The semiquantitative analysis of type I collagen immunopositive region further confirmed the above results (Figure 6(f)). The mean IOD of type I collagen in the AR administered group was significantly reduced in a dose-dependent manner compared to the model group (*P* < 0.01). It was previously reported that HMGB1 interacted with the receptor of RAGE to regulate the expression of type I collagen [17]. Therefore, we explored the effects of AR on the expression of downstream targets of HMGB1, such as RAGE, p-MEK1/2, p-ERK1/2, p-cJun, and type I collagen. Western blot method was used to detect the expression of RAGE, p-MEK1/2, p-ERK1/2, and p-cJun protein after administration of AR (Figures 6(g)–6(k)). The proteins of RAGE, p-MEK1/2, p-ERK1/2, and p-cJun were increased remarkably only in the CCl_4_-induced group compared with the control group (*P* < 0.05, *P* < 0.01). Compared with the model group, the high-dose administration group of AR significantly reduced the expression of RAGE, p-MEK1/2, p-ERK1/2, and p-cJun (Figures 6(h)–6(k), *P* < 0.05, *P* < 0.01).

## 4. Discussion

In this study, the aqueous extract of AR was used, which is consistent with the clinical use of traditional Chinese medicine. And the research results are more valuable. To determine whether AR has a protective effect on liver fibrosis, CCl_4_-induced liver fibrosis model was performed in rats. Consistent with clinical observations, AR could improve liver function to some extent to alleviate liver injury [28, 29]. In this study, the results demonstrated that AR could reduce serum markers of liver fibrosis, including COL-IV, HA, LN, and PC-III. The examination of serum levels and liver histopathology exhibited that AR showed obvious anti-liver fibrosis effect and the anti-liver fibrosis effect of AR at the dose of 10.8 g/kg is comparable to that of colchicine. In order to apply AR more scientifically, the potential mechanism of AR on alleviating liver fibrosis was further explored.

Previous reports and the results of this study confirmed that AR has definite hepatoprotective effect and can effectively improve liver fibrosis [19, 30, 31]. However, how to exert the therapeutic effect is still unclear. Previous studies have reported that AR has strong anti-inflammatory activity and can effectively reduce the expression of inflammatory factors such as iNOS, cyclooxygenase-2 (COX-2), IL-6, TNF-*α*, and IL-1*β* [32, 33]. Long-term inflammatory response is the key to the occurrence of liver fibrosis [34]. Therefore, we detected the effect of AR on the expression of inflammatory factors in liver fibrosis rats. The results showed that AR could significantly downregulate the expression of IL-1*β*, TNF-*α*, and IL-6. It suggested that AR might improve liver fibrosis by inhibiting the inflammatory response in rats.

Pathogen-free inflammatory liver disease is considered “sterile” [35]. Studies have reported that DAMP is the key to aseptic inflammation after liver injury, and HMGB1 is the key point [36]. Extracellular HMGB1 is a multifunctional cytokine, which induces inflammation by binding with specific cell surface receptor TLR4 [37]. Thus, we detected the expression of HMGB1 in rats to explore whether AR had effect on regulating HMGB1 expression via improving the inflammatory response. The results showed that the expression of HMGB1 decreased in the AR treatment group compared with the model group. The TLR4 signaling pathway is the most studied pathway. TLR4, as a pattern recognition receptor, recruits Myd88 under the action of the endogenous activator HMGB1, which causes the activation of the NF-*κ*B inflammatory pathway and promotes the transcription of inflammatory cytokines IL-1*β*, TNF-*α*, and IL-6 [38, 39]. Therefore, we detected the expression of TLR4, Myd88, and NF-*κ*B p65. The results showed that the expression levels of TLR4, Myd88, and NF-*κ*B p65 in AR treatment group were significantly lower than those in the model group. The above results suggest that AR might reduce CCl_4_-induced liver inflammatory by downregulating the expression of HMGB1/TLR4/NF-*κ*B signaling pathway.

**Figure 7:**
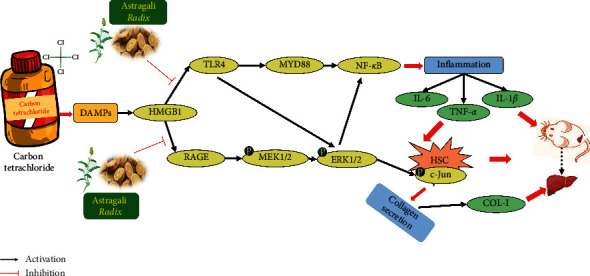
Schematic diagram of signal pathway which mediates CCl_4_-induced liver fibrosis and the ameliorative effects of AR on rat.

In experimental and human liver injury, activated HSCs (myofibroblasts) are recognized as the driving factors of liver fibrosis [40]. It has been reported that HSCs activated by inflammatory factors are the main source of myofibroblasts in the liver [34]. And HMGB1 could directly stimulate the activation of HSCs [16]. The pharmacodynamic results show that AR can effectively improve the liver fibrosis in rats induced by CCl_4_. The study also found that AR can not only downregulate the expression of HMGB1, but also reduce the transcription of inflammatory cytokine IL-1*β*, TNF-*α*, and IL-6 by regulating HMGB1/TLR4/NF-*x*B signaling pathway. Therefore, we speculate that AR can inhibit the activation of rat HSCs. The detection of *α*-SMA, a marker protein of HSCs activation, further confirmed our hypothesis: AR can significantly inhibit the activation of rat HSCs induced by CCl_4_ (Figure 7).

Liver fibrosis or scar deposition caused by chronic injury is similar in all forms of liver disease [41]. In the subendothelial space between hepatocytes and endothelial cells, fibrillar or type I collagen aggregates to replace the low-density basement membrane-like matrix containing type IV collagen. The conversion of the subendothelial matrix to fibril-rich collagen is a key event, which mediates the loss of differentiation function characteristic of progressive liver disease [42]. Myofibroblasts are the main source of type I collagen accumulated during tissue fibrosis [43]. It was found that the activation of HSCs was decreased in AR-treated group, and we speculated that the expression of type I collagen in the liver of rats would also be reduced accordingly. In addition, the latest research reports that HMGB1 activates the paracrine system and upregulates the secretion of type 1 collagen by binding to the receptor RAGE on the surface of fibroblasts, thereby triggering and/or maintaining scar formation [17]. Therefore, we detected the expression of type I collagen in the liver of rats, as well as the expression of RAGE, p-MEK1/2, p-ERK1/2, and p-c-Jun in liver tissues. The results showed that AR can significantly reduce the expression of type 1 collagen in rats, which may be related to the decrease of HSCs activation and the downregulation of collagen secretion mediated by HMGB1-RAGE-c-Jun.

## 5. Conclusion

In conclusion, biochemical tests and histological assessments were used to reveal the function of AR. The study also demonstrated that AR was potent for downregulating the expression of HMGB1/RAGE/cJun pathway and HMGB1/TLR4/NF-*κ*B pathway, thus protecting rats against CCl_4_-induced liver fibrosis. Therefore, this study will provide scientific evidence and theoretical basis for the development of AR as a potential drug candidate for liver fibrosis.

## Figures and Tables

**Table 1:** Primers sequences for RT-PCR.

Gene	Sense primer (5′-3′)	Antisense primer (5′-3′)
IL-6	AGGAGTGGCTAAGGACCAAGACC	TGCCGAGTAGACCTCATAGTGACC
TNF-*α*	GCATGATCCGAGATGTGGAACTGG	CGCCACGAGCAGGAATGAGAAG
IL-1*β*	ATCTCACAGCAGCATCTCGACAAG	CACACTAGCAGGTCGTCATCATCC
HMGB1	ACAACACTGCTGCGGATGACAAG	CCTCCTCGTCGTCTTCCTCTTCC
TLR4	GCTGCCAACATCATCCAGGAAGG	TGATGCCAGAGCGGCTACTCAG
Myd88	CGACGCCTTCATCTGCTACTGC	CCACCACCATGCGACGACAC
RAGE	CTGCCTCTGAACTCACAGCCAATG	GTGCCTCCTGGTCTCCTCCTTC
GAPDH	GTCCATGCCATCACTGCCACTC	GATGACCTTGCCCACAGCCTTG

## Data Availability

The data used to support the findings of this study are available from the corresponding author upon reasonable request.

## Abbreviations

AR: *Astragali Radix*
ALT: Alanine aminotransferase
AST: Aspartate aminotransferase
CCl_4_: Carbon tetrachloride
COL-IV: Collagen IV
DAMPs: Damage-associated molecular patterns
ECM: Extracellular matrix
HA: Hyaluronic acid
HMGB1: High-mobility group box 1
HSCs: Hepatic stellate cells
H&E: Hematoxylin-eosin
IHC: Immunohistochemistry
IOD: Integrated optical density
LN: Laminin
PCR: Polymerase chain reaction
PC III: Precollagen type III
RAGE: The receptor of advanced glycation end products
SD: Standard deviation
*α*-SMA: *α*-smooth muscle actin.
